# Follicular Dynamics and Pregnancy Rate in Nellore Heifers Submitted to Fixed-Time Artificial Insemination Protocols (FTAI)

**DOI:** 10.3390/vetsci9080377

**Published:** 2022-07-22

**Authors:** Filipe Prudente da Silva, Kedson Alessandri Lobo Neves, Francisco R. A. Correa, Lílian K. X. Silva, Helder R. Batista, Welligton C. da Silva, Nohora Mercado Caruso, Antonio Humberto Hamad Minervino

**Affiliations:** 1 Centro Universitário da Amazônia, Santarém 68010-200, PA, Brazil; prudentefilipe@gmail.com (F.P.d.S.); araujocorrear@gmail.com (F.R.A.C.); silva_lilian@yahoo.com.br (L.K.X.S.); hbatista.mv@gmail.com (H.R.B.); welligton.medvet@gmail.com (W.C.d.S.); 2 Laboratory of Animal Health (LARSANA), Federal University of Western Pará (UFOPA), Rua Vera Paz, s/n, Salé, Santarém 68040-255, PA, Brazil; kedson.neves@ufopa.edu.br; 3 Departamento Productividad e Innovación, Universidad de la Costa, CUC, Calle 58 n.55-66, Barranquilla 080001, Atlántico, Colombia; nmercado1@cuc.edu.co

**Keywords:** *Bos indicus*, reproductive efficiency, biotechnology, hormones

## Abstract

**Simple Summary:**

Reproductive biotechnologies are important strategies to enforce the production of genetically superior animals. The fixed-time artificial insemination is widely used in Brazil and it is especially important in farms located in remote areas, contributing to a better management and increase in genetics and productivity in areas such the Lower Amazon. We evaluated two different protocols of fixed-time insemination using two different hormones in the Nellore heifer raised under tropical conditions. The protocol of the fixed-time artificial insemination using equine chorionic gonadotrophin presented a better outcome in the pregnancy rate compared to the protocol using follicle stimulating hormone. Our tested protocol may be the most suitable alternative to increase conception rates in animals that are raised in an extensive system under tropical conditions in the Amazon.

**Abstract:**

This study aimed to evaluate follicular dynamics and pregnancy rates in Nellore heifers submitted to fixed-time artificial insemination (FTAI) protocols associated with equine chorionic gonadotrophin (eCG) or follicle stimulating hormone (FSH). Nellore heifers (n = 259) were used, divided into two studies. *Experiment I* evaluated the ovarian follicular dynamics in 64 Nellore heifers submitted to different FTAI protocols (n = 32/group) using either FSH or eCG. In *Experiment II*, the pregnancy rate was evaluated in 195 heifers submitted to FTAI protocols and divided into two groups: FSH (n = 97) and eCG (n = 98). In *Experiment I*, the ultrasound examination showed that the maximum diameter of the dominant and preovulatory follicles and the ovulation time were similar between the FSH and eCG groups (*p* > 0.05). However, the ovulation rate was higher in the eCG group when compared to FSH (*p* = 0.014). In *Experiment II*, females that received eCG presented a higher pregnancy rate (58.1%) when compared to FSH (40.2%) (*p* = 0.012). The use of eCG in the FTAI protocol in Nellore heifers promoted a higher ovulation rate and increased pregnancy rate and may be the most suitable alternative to increase conception rates in animals that are raised in an extensive system under tropical conditions in the Amazon.

## 1. Introduction

The reproductive efficiency of cattle herds is influenced by several factors, such as the breeding system, the breed used, the management employed on the farm, and the sanitary and nutritional status of the animals [1,2]. Despite this variety of factors that interfere in animal reproduction, the use of reproductive biotechnologies in the bovine herd has shown positive results, and it is of fundamental importance for the economic growth of national cattle breeding [3,4,5,6].

In this context, it is worth mentioning fixed-time artificial insemination (FTAI), which is a technique that eliminates difficulties faced in conventional artificial insemination, thereby removing the need for detection of estrus since it uses pharmacological protocols that allow synchronized ovulation, increases the rate of service and conception and favors the genetic improvement of the herd [7,8]. The use of hormonal protocols with progesterone and estrogens is well established in the literature [9,10,11] and allows conception rates around 50% [8,12]. FTAI protocols in *Bos indicus* postpubertal heifers have resulted in lower pregnancy rates than in anestrous cows [13], but FTAI protocols adaptations for heifers can improve pregnancy rates up to 60% or more [14,15].

Among the available hormone protocols for heifers, the use of follicle stimulating hormone (FSH) and equine chorionic gonadotropin (eCG) are widely used in Brazil [16]. FSH is a glycoprotein produced and secreted by gonadotrophic cells, which are present in the anterior region of the pituitary gland [17]. It has been widely used in FTAI protocols because it stimulates follicular development and maturation [18]. The function of FSH is to stimulate the production of estrogens by the ovarian follicles through the activity of the aromatase enzyme. Dominant follicles, which have a diameter of 4 to 9 mm, require high concentrations of FSH. The follicle stimulating hormone interacts with receptors and triggers important intracellular reactions for the production of steroid hormones, since follicles grow under FSH action. Subsequently, by reaching adequate FSH/LH concentrations, it establishes the levels necessary for ovulation to occur [10].

Differently from FSH, equine chorionic gonadotropin (eCG) is naturally produced in the mare’s endometrial calyces during gestation (40 to 130 days). Its use in FTAI programs in cattle can improve fertility rates, especially in heifers and in recent postpartum cows [19], with a recent study showing a positive effect in pregnancy rates [16]; however, some studies have not been able to demonstrate the effectiveness of eCG in FTAI protocols for beef heifers [20]. Equine chorionic gonadotropin can bind to the FSH and luteinizing hormone (LH) receptors, possessing activities directly related to follicular development; therefore, it can provide follicular growth and maturation, and consequently, ovulation [21]. In addition, eCG can increase the preovulatory follicle diameter during FTAI, improve ovulation rate, and elevate plasma progesterone concentrations in the luteal phase [19]. Both hormones, FSH and eCG, can be used in FTAI protocols with a single dose on the day of the progesterone intravaginal device removal [16].

In this study, we adopted the hypothesis that the adoption of the hormonal FTAI protocol using eCG would promote an increase in the ovulation and pregnancy rates in Nellore heifers raised under pasture conditions in the Amazon, compared to the FSH protocol. Given the context, the present study aimed to evaluate the follicular dynamics and pregnancy rate in Nellore heifers subjected to FTAI protocols associated with eCG) or FSH.

## 2. Materials and Methods

All animals were managed on a commercial farm during routine management procedures and FTAI. Cows were treated according to best practices in veterinary care and animal welfare. The present study was approved by the Ethics Committee on Animal Use of the Institute of Biodiversity and Forestry, Santarém, Brazil (CEUA/IBEF) under protocol number 04.002/13).

### 2.1. Study Site

The experiments were conducted on a farm in the municipality of Mojuí dos Campos, Pará, Brazil (2°28′ and 4°23′ S and 54°31′ and 55°40′ W). The climate is hot and humid (Am4) with annual rainfall between 1900 to 2100 mm, an average annual air temperature of 25.6 °C, and relative humidity ranging from 84 to 86% [22]. The rainiest quarter occurs between the months of February and April and it is the least rainy between the months of August and October [22]. The experimental period lasted from May 2017 to March 2018.

### 2.2. Experimental Animals

The females were healthy, without any reproductive alterations, cyclic, with a mean age of 21 ± 2 months, mean weight 340 ± 1.9 kg, mean body condition score (BCS) 3.1 (ranked from 1 to 5, 1 = firm and 5 = obese) [23]. The heifers were kept at pasture with brachiaria grass (*Brachiaria brizanhta* cv. Marandu) and had free access to water and mineral mixture. The females were randomized, considering the BCS to form the experimental groups. Nellore heifers (n = 259) were divided into two experiments. *Experiment I* evaluated follicular dynamics using either FSH (FSH group, n = 32) or eCG (eCG group, n = 32) FTAI protocols. *Experiment II* evaluated the pregnancy rate of heifers submitted to FTAI with a protocol containing FSH (n = 97) or eCG (n = 98). A limitation of this study was the fact that the reproductive tract score was not performed at the time of the randomization of the experimental groups; however, since it was a homogeneous group of animals from the same property, this limitation did not affect the validity of the results obtained.

### 2.3. Experimental Design

**Figure 1:**
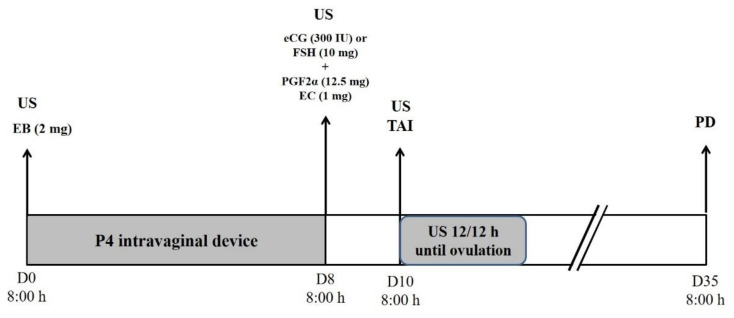
Hormonal protocol scheme used in the study. eCG = equine chorionic gonadotropin; FSH = follicle stimulating hormone; EB = estradiol benzoate; PGF2α = prostaglandin; EC = estradiol cypionate; TAI = fixed-time artificial insemination; PD = pregnancy diagnosis; US = ultrasound evaluation (performed only in *Experiment I*).

In *Experiment I*, the females were subjected to two different hormonal FTAI protocols (Figure 1). On day 0 (D0) at 8 h an intravaginal progesterone device (CIDR^®^) was introduced, then 2.0 mg of estradiol benzoate (Gonadiol^®^, Zoetis, Campinas, Brazil) was administered intramuscularly (IM). On day eight (D8) at 8 h, the progesterone implant was removed and administered by IM 2.5 mL of PGF2α (Lutalyse^®^) and 1 mg of estradiol cypionate (ECP^®^). At this time, heifers randomly received 300 IU of eCG (Novormon^®^) or 10 mg of FSH (Folltropin-V^®^) via IM. All hormones were from Zoetis (Campinas, Brazil) with exception of FSH which was bought from Vetoquinol Saúde Animal Ltd. (São Paulo, Brazil). On day ten (D10) from 8 am, the FTAIs were performed. In *Experiment II*, we evaluated the pregnancy rate of Nellore heifers subjected to FTAI with the same protocols containing FSH or eCG (Figure 1).

### 2.4. Follicle Dynamics Evaluation—Experiment I

For assessing follicular dynamics, females in *Experiment I* were divided into two groups: FSH Group (n = 32) or eCG Group (n = 32). An ultrasound (US) device (Mindray^®^, DP-10 VET) equipped with a 7.5 MHz linear transducer (Nanshan^®^) was used to assess the presence of corpus luteum of heifers on day zero (D0), and the diameter of the dominant follicle on day eight (D8) and of the pre-ovulatory follicle on day ten (D10), and thereafter every 12 h until ovulation or until 96 h after FTAI. Ovulation time was defined when detected by US. Ovulation rate considered the heifers that ovulated and failed to ovulate within the 96 h post-FTAI interval, when the US exam was performed sequentially.

### 2.5. Evaluation of the Pregnancy Rate—Experiment II

To evaluate the pregnancy rate, 195 heifers that were subjected to hormonal protocol containing FSH (n = 97) or eCG (n = 98) were used. The pregnancy diagnosis was performed by US 35 days after the FTAI, and the presence of a viable embryo with a heartbeat indicated pregnancy.

#### Statistical Analysis

The analyses were performed separately, considering experiments I and II. The data were analyzed by the Chi-square test, used to determine whether there was a significant influence of the treatments (eCG or FSH) on ovulation and conception rates. Additionally, in *Experiment I*, the data referring to the number of hours to ovulation were submitted to the Kolmogorov–Smirnov test, and since they presented a non-Gaussian distribution, they were compared by the Kruskal–Wallis test to assess whether the treatment had interference in the ovulation moment. The significance level adopted was 5% and the analyses were performed in statistical software (Minitab 18, Minitab Inc., La Jola, CA, USA).

## 3. Results and Discussion

Table 1 shows the results of the two experiments and the differences between the eCG and FSH groups. In *Experiment I*, the maximum diameter of the dominant and pre-ovulatory follicle and the ovulation time were similar between the FSH and eCG groups (*p* > 0.05). However, the ovulation rate was higher in the eCG group (87.5%) when compared to the FSH group (78.1%) (*p* = 0.014). Similar results were observed in a study with Nellore cows using eCG and FSH, where the ovulation rate was lower in the FSH [24]. This is probably related to the short half-life of FSH, which is around 5 h compared to eCG, which has a long half-life of around 46 h, maintaining a high concentration for a longer time [7,25]. Previous studies have also demonstrated an increased ovulation rate in Nellore heifers treated with eCG compared to FSH [3].

The eCG supplies the lack of LH and provides the follicle with the ability to develop until the pre-ovulatory phase, which contributes to increase the production of follicular estrogen. In this way, the gradual increase in plasma estrogen causes positive feedback in the hypothalamus, determining the pre-ovulatory peak of LH [26]. Nevertheless, in the present study there was no difference in relation to the diameter of the ovulatory follicle (OF) or dominant follicle (DF) and the group that received FSH had a higher ovulation rate. In a study evaluating the effect of eCG (400 IU) in FTAI protocols in cyclic and acyclic Nellore heifers, there was a significant effect on FD diameter (10.6 ± 0.2 mm; *p* = 0.003) and ovulation rate (94.3 and 73.6%; *p* = 0.006). [19].

There were no differences (*p* > 0.05) in the diameters of the dominant and pre-ovulatory follicles between the FSH and eCG groups, which may indicate that FSH promotes an adequate development in the final growth of the follicle in Nellore heifers. However, other studies have shown results indicating that FSH reduced follicular development compared to eCG when used in FTAI protocols in heifers [18].

Regarding pregnancy rate, it was found that females submitted to the pharmacological protocol with eCG showed better results when compared to FSH. The eCG positively influences the pregnancy rate, providing an increase in the fertility of the bovine herd [9,27]. Similar results to those obtained in the present study have already been described, with a pregnancy rate of 58.2% (71/122) using eCG in cyclic Nellore heifers [25] and an increase in pregnancy rate when using eCG in FTAI protocols [28].

## 4. Conclusions

Both of the treatments used efficiently induced follicular synchronization in Nellore heifers, which can probably be related to their good body condition score. The use of eCG in the FTAI protocol in Nellore heifers promoted a higher ovulation rate and increased pregnancy rate, and may be the most suitable alternative, aiming to raise the conception rates in animals bred in an extensive system under tropical conditions in the Amazon.

## Figures and Tables

**Table 1:** Mean and standard deviation and comparative analysis of productive and reproductive variables of Nellore heifers subjected to fixed-time artificial insemination protocol using eCG or FSH.

Variables	FSH	eCG	*p*
*Experiment I*			
N	32	32	NA
B.C.S. ^§^	3.11 ± 0.21	3.10 ± 0.17	0.957
Diameter DF at D8 (mm) ^§^	10.5 ± 2.76	10.14 ± 2.92	0.915
Diameter OF (mm) ^§^	12.75 ± 2.70	12.14 ± 3.5	0.882
Ovulation time * (h) ^§^	77.76 ± 9.24	74.48 ± 19.34	0.212
Ovulation rate ^†^	78.12%	87.5%	0.014
*Experiment II*			
N	97	98	NA
Pregnancy Rate ^†^	40.2%	58.1%	0.012

N = number. DF = dominant follicle. B.C.S. = body condition score. OF = ovulatory follicle. NA = not available. FSH = follicle stimulating hormone. eCG = equine chorionic gonadotropin. D = day. ^§^ Comparison between groups by Kruskal–Wallis test. Comparison between groups by Chi-square test. * Ovulation time (in hours) related to the time of intravaginal implant removal. ^†^ Refers to the percentage of heifers with ovulation detected within the 96 h period of US evaluation after FTAI.

## Data Availability

The raw data is available upon request.
